# Anterior percutaneous full-endoscopic transcorporeal decompression of the spinal cord for single-segment cervical spondylotic myelopathy: The technical interpretation and 2 years of clinical follow-up

**DOI:** 10.1186/s13018-019-1474-5

**Published:** 2019-12-23

**Authors:** Weijun Kong, Zhijun Xin, Qian Du, Guangru Cao, Wenbo Liao

**Affiliations:** 1 Department of Orthopaedic, The Second Affiliated Hospital of Zunyi Medical University, 1 Xinpu Rd, Zunyi, 563000 Guizhou China; 2 Department of Spine Surgery, The First Affiliated Hospital of Zunyi Medical University, Zunyi, 563000 Guizhou China

**Keywords:** Percutaneous, Transcorporeal, Cervical spondylotic myelopathy, Full-endoscopic, Decompression of spinal cord, Efficacy

## Abstract

**Background:**

ACDF is the standard procedure for treatment of cervical spondylotic myelopathy (CSM), but a long-term follow-up has been revealed some associated complications of swallowing discomfort, displacement of the fusion device, and accelerated degeneration of the adjacent segment.

**Objective:**

To evaluate the clinical outcomes of anterior percutaneous full-endoscopic transcorporeal decompression of the spinal cord (APFETDSC) for single-segment CSM and to analyze the clinical efficacy, surgical characteristics, and complication prevention.

**Methods:**

A total of 32 patients who underwent APFETDSC for single-segment CSM from Aug. 2015 to Apr. 2017 were reviewed. Operating time, time of walking out of bed postoperation, length of hospitalization, complications, neck pain visual analog scale (VAS), and Japanese Orthopaedic Association Score (JOA) were evaluated. Measurement of intervertebral height (HI) of surgical segments on cervical neutral X-ray, Harrison’s method was used to measure cervical spine angle (CSA).

**Results:**

The operation time was 103.3 ± 12.95 min, time of walking out of bed after surgery was 19.81 ± 4.603 h, the length of postoperative hospital stay was 57.48 ± 19.48 h. The postoperative neck pain VAS and JOA were significantly improved compared with preoperation(*p* < 0.001). The postoperative HI was statistical significance decreased compared with preoperation(*p* < 0.001), but the HI reduction was less than 0.5 mm, without adverse clinical symptoms. The postoperative CSA was significantly improved compared with preoperative(*p* < 0.001). The excellent and good rate was 87.5%, and the JOA improvement rate was 75.52 ± 11.11%. There was no cervical instability, vertebral fracture, wound infection, and other complications.

**Conclusions:**

APFETDSC is a safe and effective minimally invasive technique with small auxiliary injuries for single-segment CSM while avoiding the sequelae of ACDF. Its short-term clinical efficacy was good and no significant effect on cervical stability.

## Introduction

Cervical spondylotic myelopathy (CSM) is a series of clinical symptoms and signs that result from cervical spine degeneration and compression of the spinal cord, and patients are primarily characterized by numbness in the upper extremities or lower limbs, thoracic fasciculation, clumsy hands, feelings of walking on cotton, and even sphincter dysfunction [1]. Regarding the treatment of CSM patients, anterior cervical discectomy and fusion (ACDF) was adopted in 1950, and this procedure was widely accepted and is now the standard procedure for the treatment of CSM [1, 2]. Although good interbody fusion is obtained, this procedure can lead to changes in the biomechanics of the cervical vertebrae, and there is a shortage of adjacent segment degeneration acceleration, and complications of dysphagia, dysphonia, hematoma, and so on arose due to the use of a front plate and the fusion of the local segments [3, 4].

Due to the technique of spinal endoscopy for the treatment of spinal degenerative diseases presents superior characteristics compared with open surgery because of the provision of a clear view, minimal trauma, ability to excise targeted prominent lesions, and significantly reduced perioperative complications [5, 6]. Spinal endoscopy has been widely used in the treatment of lumbar degenerative diseases, and endoscopic surgery for cervical degeneration diseases is a hotspot in spine surgery [7–9]. Endoscopic discectomy via the transdisc approach for cervical disc herniation has been used in the clinic, but further damage to the degenerated disc via the transdisc approach is inevitable and accelerates the loss of intervertebral height, even can cause spontaneous bone fusion in the later stage [10, 11]. George et al. reported the transcorporeal method to dispose cervical spondylosis [12]. Sakai T et al. reported on the use of the anterior opening transcorporeal approach to establish tunnel with microscopically assisted excision of the protruding discs and acquired good therapeutic effect [13]. We reported a cohort study using percutaneous full-endoscopic transcorporeal cervical discectomy [14]. This procedure focuses on soft prominent disc tissue, which does not elucidate in effective decompression of the spinal cord by disc-osteophytes, with the further application of spinal endoscopy in the treatment of cervical spondylosis. This study utilized the natural space between the anterior visceral sheath of the cervical spine and the vascular sheath by APFETDSC for single-segment CSM. Whether segmental instability and acceleration of cervical degeneration can occur on postoperative of APFETDSC has become the focus of specialists. This study aimed to analyze the early clinical outcomes of APFETDSC cases and to evaluate the clinical safety and effects on cervical spine stability.

## Methods

### General information

This is a single-center clinical retrospective study. From Aug. 2015 to Apr. 2017, 32 patients with single-segment CSM underwent spinal cord decompression treatment with the anterior percutaneous full-endoscopic transcorporeal procedure: 18 males and 14 females of 59.25 ± 6.849 years (51~73 years). The disease course was 8.766 ± 4.887 months (3–21 months). The distribution of clinical symptoms along with the type of lesion and their level was shown in Table 1. The magnetic resonance imaging (MRI) and computerized tomography (CT) scans to identify the characteristics and exact location of the lesion. The compression of the spinal cord was mainly attributable to single-segment protruding discs-osteophytes complex [15]. And CT scan was utilized for preoperative measurement of the depth and direction of the tunnel trajectory. Patients were excluded for cervical instability, calcification of the posterior longitudinal ligament, yellow ligaments or simple posterior compression, and compression of the two or more segments spinal cord; Cases of coagulant dysfunction, intervertebral infection, cervical malformation, and anterior cervical surgery history were excluded. The study was performed after obtaining due permission from the local institutional review board as well as informed consent from the patients.

**Table 1:** Clinical features and affected level (*n* = 32)

Demographics	Number of patients (%)
Preoperative clinical features	
Neck pain	32 (100%)
Inflexible hand movements	26 (81.25%)
Unsteady gait	8 (25%)
Sensory numbness	28 (87.5%)
Motor weakness	16 (50%)
Tendon reflex abnormalities	20 (62.5%)
Positive Hoffman’s sign	24 (75%)
Level of pathology	
C6–7	3 (9.4%)
C5–6	15 (46.9%)
C4–5	12 (37.5%)
C3–4	2 (6.2%)

The spinal endoscopy system (SPINENDOS GmbH., Germany) comprised a 30^°^-angled scope, a 4.3-mm working channel with a water irrigation system, a 6.9-mm outer sheath, and related surgical instruments, a gimmi-SPINENDOS digital camera system. And a low-temperature radiofrequency system (ArdthroCare Co., California, USA).

### Surgical methods

**Fig. 1.**
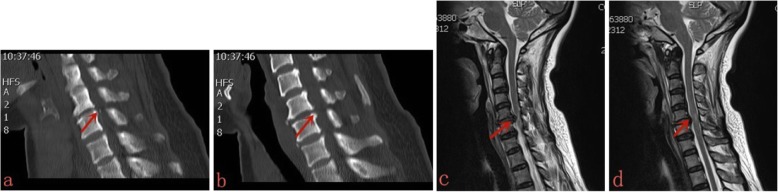
Preoperative CT images indicating that the cervical 5/6 intervertebral disc-osteophyte complex was formed(**a**, **b**); Preoperative MRI images indicating that the spinal cord was clearly compressed by disc-osteophyte complex (**c**), and the abnormal signal was showed (**d**)

**Fig. 2.**
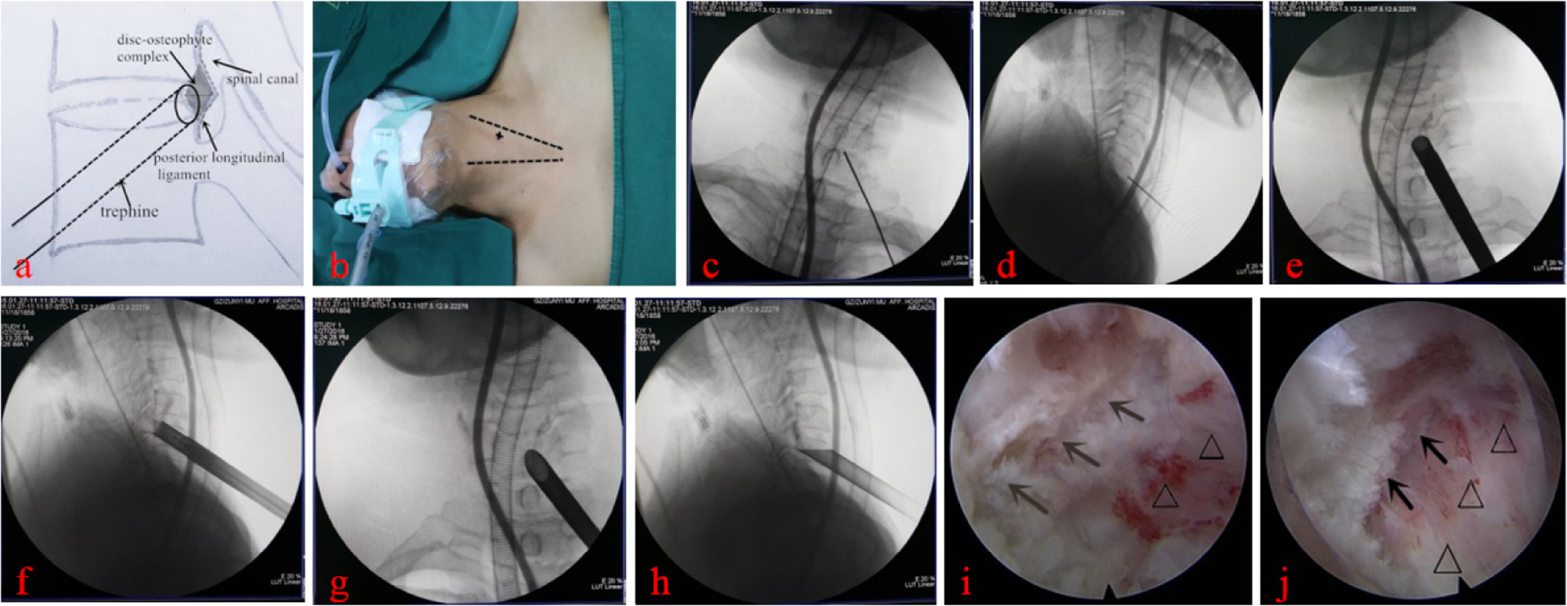
Schematic diagram of operation channel planning established via vertebral body before surgery (**a**). Preoperative gastric tube indwelling, puncture site localization, and surface marking schematic diagram (**b**). The position of the positioning needle was determined by the anterior-posterior(AP)and lateral C-arm X-ray perspective(**c**, **d**).The trephine were used to establish the bone channel and C-arm fluoroscopy was used to monitor the direction and depth of the trephine (**e**, **f**). AP and lateral C-arm X-ray fluoroscopy show the location of the work sleeve (**g**, **h**). In the intraoperative endoscopic image, the arrow indicates the intervertebral disc-osteophyte complex, and the triangle indicates the posterior longitudinal ligament(**i**). Intraoperative images of decompression of the spinal cord under endoscope, arrows indicating the posterior margin of the upper vertebral body, and triangles indicating the dura sac (**j**)

The operations were performed by the corresponding author. All patients had gastric tube inserted prior to the surgery and were administered general anesthesia with endotracheal intubation, and the patient was placed in the supine position and kept the neck slightly overstretched. Downward traction was applied to the upper limbs to avoid the blocking of the lower cervical intervertebral space on X-ray. Fifteen milligrams of iodohydride was injected into the gastric tube to show the position of the esophagus under the c-arm perspective and the skin puncture point was marked on the far side of the esophagus. Strict surgical disinfection was performed, and sterile waterproof coating covers were used in the surgical area to avoid flushing the sterile dressing. According to preoperative CT and MRI, the locations of the disc-osteophyte complex were identified (Fig. 1a–d), and the trajectory of the osseous channel was designed (Fig. 2a). The puncture point was located in the lower part of the lower vertebral body under the section of the responsible segment. Using the “two-fingering” technique at the puncture point, the endotracheal sheath of the esophagus and the vascular nerve sheath were pushed aside to touch the front of the vertebral body, and a puncture-safe area was created with the skin pressed to the vertebral body [16, 17]. A puncture needle was inserted between the fingers. The position of the puncture point and the direction of the guide needle in the vertebral body were established according to the C-arm X-ray perspective (Fig. 2c, d). When the guide needle properly inserted the vertebral body fixation, and the expansion sleeve (*D* = 6.3 mm) was inserted along the guide needle, the tube was slowly rotated and placed in the protective casing (*D* = 8.5 mm). Under the protection of the trephine casing, according to the target of disc-osteophyte complex, the bone tunnel was gradually established. The bone tunnel continued until the trephine penetrated the posterior margin of the vertebral body in the C-arm monitoring perspective (Fig. 2e–f), and the trephine was properly shaken to remove the vertebral bone. The trephine protective casing was replaced with a work casing (7.6 mm) (Fig. 2g, h). The endoscopic operating system was inserted through the casing, and the endoscopic decompression procedure began. Continuous saline irrigation was used to maintain a clear view during the operation. Various types of medullary forceps were used to remove the protruding disc tissue. A high-speed diamond burr and rongeur were used to clear up the disc-osteophyte complex (Fig. 2i), and epidural capsule decompression and pulsation were acquired (Fig. 2j). Hemorrhaging of the posterior longitudinal ligament or epidural was stopped with radiofrequency hemostasis. When no active bleeding was observed, the work casing was stepped out of to the front of the vertebral body. The work casing was removed, a drainage tube was not required due to no active bleeding, and the incision was sutured and covered with a sterile dressing to complete the procedure. On the first postoperative day, antibiotics, hormones, and mannitol were administered, a neck protection collar was applied, and the patient walked away from the bed. The patients were discharged 2–4 days after surgery, wore the neck collars and oral administration of mecobalamine and B-complex vitamins improve neurological function for 4–6 weeks.

**Fig. 3.**
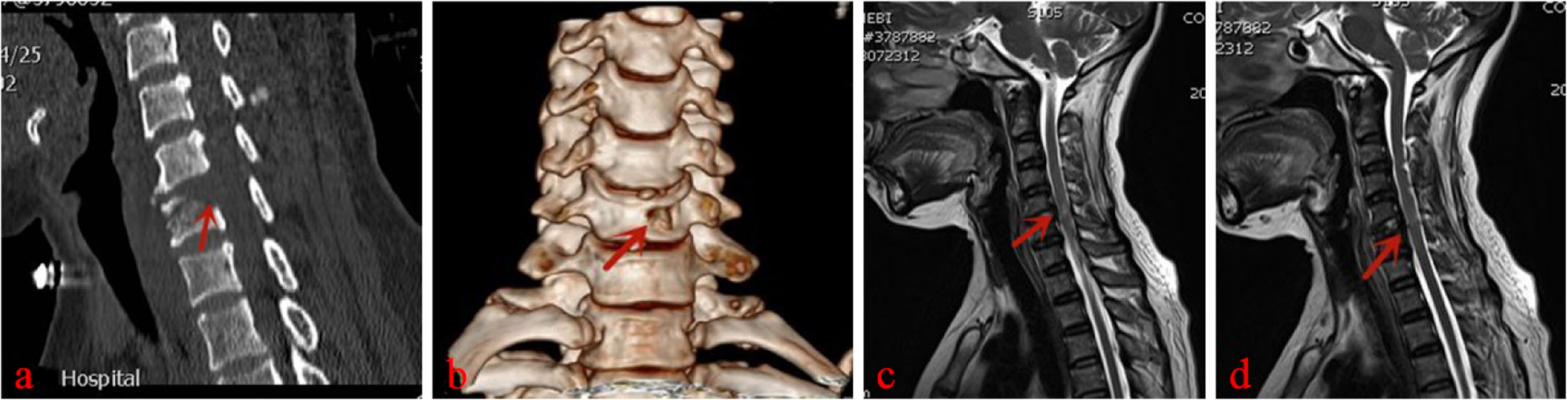
Postoperative CT showing that the disc-osteophyte complex had been removed, three-dimensional reconstruction showing the complete vertebral body tunnel, the vertebral body height is not lost, and no fracture occurs (**a**, **b**). The spinal cord decompression was sufficient on MRI examination(**c**, **d**)

**Fig. 4.**
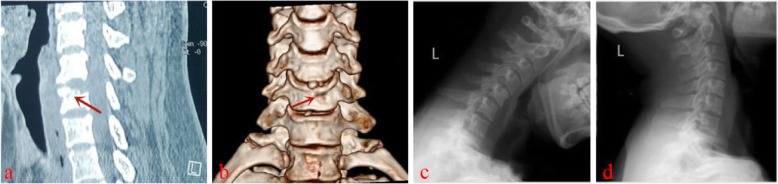
A 6-month postoperative CT examination revealed that the bone tunnel had healed and no fracture occurs (**a**, **b**). The last follow-up X-ray radiographs in flexion and extension positions of cervical spine(**c**, **d**) showed that the physiological curvature of the cervical spine remained good and there was no cervical spine instability

Typical case: Male, 51 years old, neck pain accompanied by numbness, and weakness of limbs, with the left side as the focus, C5~6 disc-osteophyte complex protrudes into the spinal canal, resulting in spinal stenosis and compression of the spinal cord (Figs. 1, 2, 3, 4).

### Evaluation indicators and methods

Operative time, surgical complications (spinal cord injury, dural injury, esophageal vascular injury, hematoma, wound infection, etc.), postoperative time of getting out of bed and postoperative hospitalization were recorded. Visual analog scale (VAS) was used to evaluate neck pain and Japanese Orthopaedic Association Score (JOA) to evaluate clinical neurological function. The degree of spinal decompression was objectively assessed by postoperative cervical MRI, and vertebral structure was assessed by CT examination. The HI between the upper and lower endplate center of the operative segment was measured by neutral cervical X-ray. CSA was measured using Harrison’s method [18]: an acute angle formed by the intersection of C2 and C7 posterior vertebral tangent lines. The patients in this study were followed up at the outpatient clinic at 1 week, 6 months, and 24 months after surgery. Methods of follow-up: VAS and JOA scores were performed, and modified MacNab standard was added for the last follow-up to evaluate the excellent-good rate of treatment. X-ray plain film of anterioposterior and lateral of cervical spine was taken to measure the HI of the surgical segment and the CSA. The stability of the operative segments was observed by X-ray plain film of the hyperextension and hyperflexion of the cervical spine.

### Statistical analyses

SPSS18.0 statistical software was used for statistical analysis. Paired *t* test was used to compare the differences of HI and CSC pre- and post-operative, a positive significance level was assumed at probability of less than 0.05.

## Results

Clinical outcomes: the operations were successful in all 32 patients and clinical symptoms have been improved significantly after surgery. The operation time was 103.3 ± 12.95 min (range, 80–125 min). The time of walking out of bed after surgery was 19.81 ± 4.603 h, the length of postoperative hospital stay was 65.22 ± 16.64 h, and the mean follow-up period was 28.97 ± 3.685 months(range from 24 to 36 months). There was no obvious bleeding, no major complications of esophageal, arteriovenous, or spinal cord injuries occurred during the operations.

The changes of VAS, JOA, HI, and CSA of preoperative and postoperative in this group were shown in Table 2. Analysis results show that the postoperative neck pain VAS score was significantly reduced compared with preoperation(*p* < 0.001). The postoperative JOA score was significantly increased compared with preoperation(*p* < 0.001). The VAS score at 6 months postoperative decreased further than that at 1 week postoperatively(*p* < 0.001). The JOA score at 6 months postoperatively improved further than at 1 week postoperatively(*p* < 0.001). The VAS and JOA scores at 24 months postoperatively were not statistically significant compared with 6 months postoperatively(*p* > 0.05). The postoperative HI was statistically significant compared with preoperation(*p* < 0.001), but the HI reduction was less than 0.5 mm and without adverse clinical symptoms. The postoperative cervical curvature was significantly improved compared with preoperation(*p* < 0.001). Compared with 1 week after surgery, the six months, and last follow-up of the HI and CSC were no statistically significant(*p* > 0.05). At the last follow-up, the distribution of clinical features and treatment effect were shown in Table 3. The excellent and good rate was 87.5%, and the JOA improvement rate was 75.52 ± 11.11%; there was no significant reduction in adjacent segment intervertebral space and osteophyte hyperplasia, and no postoperative complications of postpharyngeal foreign sensation, vertebral fracture, recurrence of symptoms, and intervertebral instability were observed.

**Table 2:** The VAS, JOA,HI and CSA change pre-operative and post-operative (x ± s n = 32).

Items	Pre-o	Post-o 1 week	Post-o 6 months	Post-o 24 months
Neck VAS	4.290 ± 0.728	1.344 ± 0.466	0.469 ± 0.439	0.483 ± 0.353
JOA	7.672 ± 1.168	13.14 ± 1.172	14.56 ± 1.256	14.64 ± 1.166
HI(mm)	6.084 ± 0.275	5.975 ± 0.260	5.978 ± 0.298	5.956 ± 0.292
CSA(°)	12.41 ± 2.890	13.64 ± 2.888	13.73 ± 2.732	13.77 ± 2.720

*Pre-o* pre-operative, *Post-o* post-operative, *VAS* visual analoge scale, *JOA* japanese orthopaedic assicioation scores, *HI* height of intervetebra, *CSA* cervical spine angle

**Table 3:** Clinical features and treatment effect at last follow-up

Demographics	Number of patients (%)
Last follow-up clinical features	
Neck pain	2 (6.25%)
Inflexible hand movements	2 (6.25%)
Unsteady gait	0 (0%)
Sensory numbness	3 (9.38%)
Motor weakness	0 (0%)
Tendon reflex abnormalities	3 (9.38%)
Positive Hoffman’s sign	4(12.5%)
Excellent and good rate	
excellent	17(53.1%)
good	11(34.4%)
fair	4 (12.5%)
poor	0 (0%)

### Complications

All patients had mild intraoperative neck edema, and the edema was subsided in 2~4 h after surgery. After the removal of the casing, three cases of bleeding in the cut were observed, and the bleeding was stopped after a few minutes of compression. One patient experienced difficulty breathing due to laryngeal spasms, which was improved after the administration of a pressurized oxygen supply and control the spasms. No dysphagia, hoarseness, spinal cord injury, cerebrospinal fluid leakage, infection, or other intra- or postoperative complications occurred.

## Discussion

CSM is often caused by the compression of the anterior spinal artery with disc-osteophyte complex and resulting in varying degrees of spinal cord injury [1, 15, 19]. For patients in whom conservative treatment for more than 3 months is ineffective, surgical treatment needs to be considered. The purpose of the surgical treatment is decompression of the spinal cord [2, 20, 21]. Currently, the surgical methods of CSM primarily include ACDF and artificial cervical intervertebral disc replacement (ACDR). Due to the strict surgical indications of ACDR, its potential complications include prosthesis looseness, subsidence, vertebral periodization, late fusion, wear, and other problems [22, 23]. Although traditional ACDF can achieve good fusion, a long-term follow-up has revealed such associated complications as swallowing discomfort, foreign body sensations, hoarseness, plate looseness, displacement of the fusion device, and acceleration of the degeneration of the adjacent segment [23–26]. Hilibrand et al. reported a 2.9% annual incidence of symptomatic adjacent segments of 374 patients 10 years after receiving ACDF [27].

In recent years, with the continuous development and optimization of optical technology, channel technology, endoscopic techniques, and the improvements in spine surgery techniques, the past contraindications have become indications for the percutaneous endoscopic treatment technique. The percutaneous endoscopic technique has become the standard procedure or optimal surgery for the treatment of lumbar disc herniations [5, 6, 8, 25, 28]. Currently, the percutaneous endoscopic technique is used to treat cervical intervertebral disc herniation; the technical trauma is small and has been taken seriously by some scholars. This attention to the work casing transdisc approach is necessary because this approach can cause new damage to the disc, accelerated intervertebral degeneration, and intervertebral height reduction [9, 10, 26–29]. Therefore, transdisc approach has not been widely used in clinical practice, and so, transcorporal approach is a reasonable surgical strategy.

Most CSM cases are due to the protrusion of a cervical intervertebral disc-osteophyte complex. The symptoms of spinal cord injury are caused by the compression of the ventral side [1, 19, 22]. Effectively completing ventral decompression of the spinal cord and achieving ideal clinical effect through the posterior technique is very difficult [3, 21, 30]. Therefore, the anterior transcorporal approach with an endoscopic technique is an effective method for the treatment of CSM. To avoid iatrogenic injuries caused by transdisc approach, this study of APFETDSC demonstrated minimally invasive, direct decompression of the cervical spinal cord, maintains the physiological curvature of the cervical spine and the stability of the operative segment [16, 21, 22], and the biomechanical structure of the disc is well preserved. By applying the two-finger pressing separation method, the endotracheal sheath of the esophagus was pushed to the medial aspect, and the vascular nerve sheath was pushed to the outside [11, 17]. The skin was kept close to the vertebral body, and the position of the puncture needle was set and the injury of related viscera could be avoided. To identify the relationship between the esophagus and the puncture needle with the developing gastric tube, gradual loosening and expansion were achieved through the graded casing to safely place the working casing. Fractures were avoided because of the integrity of the vertebral body walls. A careful analysis of the compression area of the spinal cord must be performed before the operation to make detailed plans to build up the bone tunnel. The establishment of a tunnel to the spinal cord compression position in the vertebral body is the key to complete decompression of the spinal cord and guaranteeing the overall biomechanical properties of the cervical spine. To facilitate the operation, the cervical spine should be slightly overstretched. Nakai S et al. reported a complete operation by established a bone tunnel from the upper vertebral body of the lesion disc [13]. We simulated this procedure and encountered difficulties due to the blocking of the mandible. Therefore, for all cases in this group, the transcorporal approach from the lower vertebral body of the disc was selected. Careful removal of the disc-osteophyte complex tissue is necessary for fully decompression of the spinal cord and the dural sac should be fully raised and fluctuated [31, 32]. Special attention should be given to the strengths of various operating tools to avoid compression injuries to the spinal cord [33, 34].

After surgery, all patients exhibited significant improvements in limb sensation and movement disorders and no vascular and esophageal injury, and no deterioration of the spinal cord nerve function was observed. The clinical effect of this procedure was consistent with the relevant authors’ experiences [10, 35, 36]. Wen-Ching Tzaan et al. evaluated the clinical outcome data from 86 patients, and 91% of patients achieved significant postoperative clinical improvements, but there were two surgery-related complications (2%) of headache and carotid artery injury [16]. In our study, three cases of bleeding in the incision after the removal of the casing occurred, and the bleeding was stopped after a few minutes of compression. One patient had difficulty breathing and laryngeal spasms, but there was no visible swelling in the neck, and no blood were found. The spasms were improved after the administration of a pressurized oxygen supply. Incisional hemorrhage is related to capillary injury, and the bleeding can stop spontaneously. The work casing was removed when active bleeding was observed, and radiofrequency hemostatic treatment was applied. Due to the loose clearance of the cervical tissue, continuous saline irrigation was applied during the operation, which resulted in obvious cervical subcutaneous edema, but all of these occurrences were absorbed by themselves within 2 to 4 h after surgery. The patients were followed up for more than 24 months. At the last follow-up, there was no significant reduction in adjacent segment intervertebral space and osteophyte hyperplasia and no obvious changes in HI and CSC, and no complications of vertebral fracture was observed. The excellent-good rate reached 87.5%. In the patients of fair efficacy, one patient was given oral medication for neck pain with numbness and discomfort at the fingertip of the upper extremity due to frequent participation in sedentary recreational activities and was followed up. One patient suffered from neck pain after neck trauma and had obvious symptoms during hyperextension and hyperflexion activities. Re-examination of X-ray showed instability of the upper adjacent segment, and the patient refused surgery and required follow-up observation. Another two cases were associated with diabetes mellitus with peripheral nerve symptoms.

All patients chose general anesthesia, which avoided nervous, anxiety, and uncooperativeness that could be associated with local anesthesia. Although the sample size of the study group was small, the results of final follow-up were encouraging. Compared with the methods of ACDF and ACDR, APFETDSC is an alternative and effective treatment for single-segment CSM after considering the patient’s condition, open and minimally invasive cognition, whether the internal fixation plants are acceptable, treatment costs, and other comprehensive factors. The successful application of this technique requires the strict selection of cases and a rigorous and meticulous surgical operation. The operator needs to have a very rich experience in open cervical spine surgery and a very skilled operative experience in lumbar endoscopy. This study goes along with some limitations. First, for patients and doctors, the deficiency of this technique is obvious radiological exposure, and surgery time is longer than open surgery. Second, the clinical efficacy of this technique also requires further long-term follow-up evaluation of many cases. In addition, the sample size was small and only addressed single-segment disc-osteophyte complex also the limitations.

## Conclusions

APFETDSC is a safe and effective minimally invasive operation technique with small auxiliary injuries for CSM while avoiding the sequelae of ACDF or ACDR. Its short-term clinical efficacy was good and no significant effect on cervical stability. But the long-term efficacy needs longer time and more cases followed up summary.

## Data Availability

The data generated or analyzed during this study are included in this published article [and its supplementary information files].

## Abbreviations

ACDF: Anterior cervical discectomy and fusion
ACDR: Artificial cervical disc replacement
APFETDSC: Anterior percutaneous full-endoscopic transcorporal decompression of the spinal cord
CSM: Cervical spondylotic myelopathy
JOA: Japanese Orthopaedic Association scores
VAS: Visual analog scale
